# Detection and characterization of extended-spectrum β-lactamases (*bla_CTX-M-1_* and *bla_SHV_*) producing *Escherichia coli*, *Salmonella* spp. and *Klebsiella pneumoniae* isolated from humans in Mizoram

**DOI:** 10.14202/vetworld.2015.599-604

**Published:** 2015-05-14

**Authors:** Iadarilin Warjri, T. K. Dutta, H. Lalzampuia, Rajesh Chandra

**Affiliations:** Department of Veterinary Microbiology, Central Agricultural University, Selesih, Aizawl, Mizoram, India

**Keywords:** *Enterobacteriaceae*, extended spectrum β-lactamases, India, Mizoram

## Abstract

**Aim:**

The present study was conducted to isolate and characterize the extended spectrum β-lactamases (ESBLs) producing enteric bacteria in human beings in Mizoram, India.

**Materials and Methods:**

Fecal samples were collected from human beings with or without the history of diarrhea from different hospitals of Mizoram. Samples were processed for isolation and identification of *Escherichia coli*, *Salmonella* and *Klebsiella pneumoniae*. All the isolates were subjected to antibiotic sensitivity assays. Phenotypically, ESBLs production ability was determined by double discs synergy test (DDST) method. ESBLs producing isolates were subjected to polymerase chain reaction (PCR) for detection of ESBLs genes. Plasmids were cured by acridine orange. Transfer of resistance from a donor to recipient strains was done by *in vitro* horizontal method.

**Results:**

A total of 414 enteric bacteria were isolated from 180 fecal samples (113 were from diarrheic patients and 67 were from non-diarrheic patients), of which 333 (80.44%), 52 (12.56%), and 29 (7.00%) were *E. coli*, *K. pneumoniae* and *Salmonella* spp., respectively. Double discs synergy test (DDST) exhibited 72 (21.62%) *E. coli*, 12 (23.08%) *K. pneumoniae* and 4 (13.79%) *Salmonella* spp. were ESBLs producers. Altogether, 24 (13.04%) isolates were found to be positive for at least one resistance genes under this study. A total of 36 (8.70%) *E. coli*, 4 (0.97%) *K. pneumoniae* and 2 (0.48%) *Salmonella* spp. were found to be positive for *bla_CTX-M-1_* gene by PCR. Similarly, 5 (1.21%) *E. coli* and 4 (0.97%) *K. pneumoniae* isolates were found to be positive for *bla_SHV_* gene. A total of 3 (0.72%) *K. pneumoniae* isolates were recorded as positive for both *bla_CTX-M-1_* and *bla_SHV_* genes. All the isolates were carrying plasmids ranging between 0.9 kb and ~30 kb. The resistance plasmid could not be transferred to a recipient by *in vitro* horizontal gene transfer method.

**Conclusion:**

ESBLs producing enteric bacteria are circulating in human population in North Eastern Region of India. Indiscriminate use of antibiotics should be avoided to control the menace of multidrug resistance bacteria in the environment, animals, and human beings.

## Introduction

Extended-spectrum beta-lactamases (ESBLs) are enzymes that confer resistance to penicillins, cephalosporins of the first, second, and third generations and aztreonam via hydrolysis of the antibiotics [1]. These enzymes catalyze the hydrolysis of β-lactam ring of antibiotics, thereby destroying their antimicrobial property. ESBLs producing organisms are often resistant to several other classes of antibiotics, as the plasmids with the gene encoding ESBLs often carry other resistant determinants [2].

The first plasmid-mediated β-lactamase TEM-1 was originally isolated from the blood culture of a patient named Temoneira in Greece. In the early 1960s, TEM-1 being plasmid and transposons mediated has facilitated its spread to other species of bacteria. It was detected in *Klebsiella* isolated from Europe, Germany, and France in 1980, 1983, and 1985, respectively. Another common plasmid-mediated beta-lactamases is *SHV-1* (sulfhydryl variable), which is chromosomally encoded in the majority of isolates of *Klebsiella pneumoniae*, but is usually plasmid-mediated in *Escherichia coli*. Later a new family of plasmid-mediated ESBLs called CTX-M that preferentially hydrolyze cefotaxime has arisen. CTX-Ms also impart resistance against other advanced generation cephalosporins (e.g., ceftazidime) and have become the most prevalent ESBLs worldwide with many variants described [3].

*E. coli* and *K. pneumoniae* are the most common ESBL producing bacterial species. Detection of these enzymes has been observed in various other species of *Enterobacteriaceae* and *Pseudomonadaceae*. It is important to note the growing incidence of ESBLs in *Salmonella* spp. *Salmonella* strains resistant to extended-spectrum cephalosporins have been reported since the late 1980s and the number of strain types has been increasing ever since [4].

Resistant genes can spread far wider than believed by horizontal gene transfer mechanisms like conjugation, transformation, and transduction. Such gene transfer mechanisms allow mobilization of specific DNA fragments from one region to another, from plasmid to plasmid, from chromosome to chromosome and between plasmids and chromosomes. Plasmid-mediated diffusion of beta-lactamase is of great concern and contributes to the enormous spread of this kind of enzymes throughout the microbial world. Antibiotic resistance has been noted in human patient as a serious problem in intensive care units. The spread of ESBL-producing bacteria has been strikingly rapid worldwide. The third generation cephalosporins have been used in majority of patients and resistance to these antibiotics is well documented. Nowadays, ESBLs-production is considered one of the main β-lactam resistance mechanisms [5].

As very little information is available on the prevalence of ESBLs producing enteric bacteria in humans in this region, the current study was undertaken to know the prevalence of ESBLs producing *Enterobacteriaceae* in different hospitals in Mizoram.

## Materials and Methods

### Ethical approval

The work has been carried out after obtaining the approval from the Ethical Committees of the College of Veterinary Sciences and Animal Husbandry, Central Agricultural University, Selesih, Aizawl, Mizoram and all the hospitals of Mizoram.

### Bacterial isolates

Samples were collected from patients of either sex of all age groups with or without the history of diarrhea from different hospitals located in Mizoram. A total of 180 fecal samples were collected (113 were from diarrheic patients and 67 were from non-diarrheic patients) during the study. All the samples were collected using a sterile cotton swab and transported to the laboratory under the cold chain.

For isolation of *E. coli* and *K. pneumoniae*, the collected fecal samples were inoculated on MacConkey’s agar and single colonies were selected and confirmed by standard bacteriological technique. For isolation of *Salmonella* spp., samples were enriched in Selenite F broth, and streaked on *Salmonella* Shigella Agar plate. Pure colonies were then selected and identified as per standard bacteriological techniques.

### Antimicrobial susceptibility testing

Antimicrobial susceptibility test was done on Mueller-Hinton agar plate as per the recommendation of Clinical Laboratory Standard Institute [1] using the following commercially available antibiotics disc: ceftriaxone (30 mcg), ceftazidime (30 mcg), cefixime (30 mcg), cefotaxime (30 mcg), cefuroxime (30 mcg), cefalexin (30 mcg), cefpodoxime (10 mcg), ampicillin (10 mcg), ticarcillin (75 mcg), piperacillin (10 units), amoxicillin (30 mcg), aztreonam (30 mcg), imipenem/cistatin (10/10 mcg), streptomycin (10 mcg), and Nalidixic acid (30 mcg).

### Double disc synergy test

The screening for ESBLs production was carried out using cefotaxime (30 mcg) and ceftazidime (30 mcg) alone as well as cefotaxime/clavulanate (30/10 mcg) and ceftazidime/clavulanate (30/10 mcg) combination as per the recommendation of CLSI [1].

### Polymerase chain reaction (PCR) amplification of *bla_CTX-M-1_* and/or *bla_SHV_* genes

Bacterial lysate was prepared from all the suspected ESBLs positive isolates and were tested for the presence of *bla_CTX-M-1_* and/or *bla_SHV_* genes by PCR assay using specific primers [6] (Table-1). PCR was carried out in a 0.2 ml thin-wall PCR tube using the bacterial lysate as template DNA with a final volume of 25 µl containing ×1 buffer, 1.5 mM MgCl_2_, 200 pM of each oligonucleotide primers, 200 µM of each dNTPS, 1 U of Taq polymerase, and 4.0 µl DNA lysate. PCR was carried out in a thermal cycler and the cycling condition was: initial denaturation at 94°C for 5 min followed by 30 cycles of amplification with denaturation at 94°C for 1 min, annealing at 54°C for 1 min, and extension at 72°C for 1 min, ending with a final extension at 72°C for 5 min.

**Table-1:** Details of the oligonucleotide primers used in the present study.

Primer name	Sequences (5` -3`)	Expected amplicon size (bp)	References
*bla_CTX-M-1_*	F- 5’-GACGATGTCACTGGCTGAGC3’ R 5’ AGCCGCCGACGCTAATACA-3’	499bp	[6]
*bla_SHV_*	F 5’ GGGTTATTCTTATTTGTCGC - 3’ R- 5’-TTAGCGTTGCCAGTGCTC - 3’	930bp	

### Extraction of genomic and plasmid DNA

The chromosomal DNA and plasmid DNA was extracted [7] from all the isolates harboring the ESBLs genes. PCR was performed using the plasmid and chromosomal DNA separately following the above-mentioned settings to find out the location of the target genes.

### Curing of plasmid

All the isolates, carrying *bla_CTX-M-1_* and/or *bla_SHV_* genes in their plasmid were subjected to curing using acridine orange [6]. In brief, 0.2 ml of overnight culture was inoculated in 5 ml LB broth containing different concentrations (2.5 mg/ml, 2.0 mg/ml, 1.5 mg/ml, 1.0 mg/ml, 0.5 mg/ml, 0.25 mg/ml, 125 µg/ml, and 62.5 µg/ml) of acridine orange. Positive control contained only cells without acridine orange, while negative control contained only acridine orange without cells. All the tubes were incubated (in dark) at 37°C overnight. Next day tubes containing the highest concentration of acridine orange showing growth were selected and loop-full was streaked on MacConkey’s agar plates and incubated overnight.

### Horizontal gene transfer

The ability of transfer of antibiotic resistance genes within *Enterobacteriaceae* group of bacteria was recorded by *in vitro* conjugation study. *Salmonella enteritidis* (ATCC 13076) was used as recipient strain. The recipient strain was sensitive to ampicillin, gentamicin, ofloxacin, cefixime, tobramycin, nalidixic acid, kanamycin, ceftriaxone, and cefotaxime and was also not carrying *bla_CTX-M-1_* and/or *bla_SHV_* genes in its plasmid as confirmed by PCR. *In vitro* mating experiments were performed by broth mating, filter paper mating, and plate mating [6]. Trans-conjugants were selected on MacConkey’s agar containing cefotaxime @ 2 µg/ml and ceftriaxone @ 5 µg/ml. Donor and recipient strains were grown separately in antibiotic-free medium as well as an antibiotic medium as control. Selected trans-conjugants were further characterized for their antimicrobial susceptibility, ESBLs phenotype, and presence of *bla_CTX-M-1_* and/or *bla_SHV_* genes by PCR.

## Results

Of the 180 fecal samples, a total of 414 enteric bacteria were recovered, of which 333 (80.44%), 52 (12.56%), and 29 (7%) were *E. coli*, *K. pneumoniae* and *Salmonella* spp., respectively. By DDST test, a total of 88 (21.26%) were confirmed as ESBL producers, of which 72 (21.62%) were *E. coli*, 12 (23.08%) were *K. pneumoniae*, and 4 (13.79%) were *Salmonella* spp. (Table-2).

**Table-2:** Details of specimens, isolation of enteric bacteria, and detection of ESBLs production by the isolates.

Total number of samples		Name of the bacterial isolates	Total number of isolates		Suspected for ESBLs producer by initial screening		Confirmed as ESBLs producer by DDST method	
			
Diarrheic	Non-diarrheic		Diarrheic	Non-diarrheic	Diarrheic	Non-diarrheic	Diarrheic	Non-diarrheic
113	67	*E. coli*	216	117	113	63	61	11
		*K. pneumoniae*	29	23	22	12	11	1
		*Salmonella* spp.	21	8	9	3	4	0
		Total	266	148	144	77	76	12

ESBLs=Extended spectrum β-lactamases, *E. coli=Escherichia coli, K. pneumonia=Klebsiella pneumonia*

**Figure-1:**
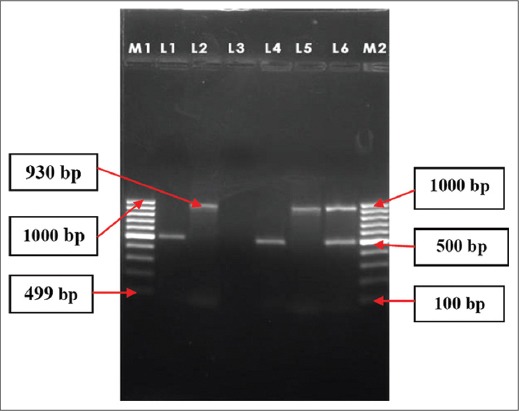
Multiplex PCR assay for detection of bla_CTX-M-1_ and bla_SHV_ genes in bacteria isolated from human beings in Mizoram. Lane M1 and M2: 100 bp DNA ladder, Lane 1: positive control for bla_CTX-M-1_ (499 bp), Lane 2: positive control for bla_SHV_ (930 bp), Lane 3: negative control, Lane 4: sample no. IEC-22 (499 bp), Lane 5: sample no. IKP-114 (930 bp), Lane 6: sample no. IKP- 94 (499 bp and 930 bp). IEC: *Escherichia coli* isolates, IKP- *Klebsiella pneumoniae* isolates.

Of the 88 phenotypically ESBLs positive isolates, 54 (13.04%) isolates carried at least one ESBLs genes tested under this study, of which 41 (9.90%) *E. coli*, 11 (2.66%) *K. pneumoniae* and 2 (0.48%) *Salmonella* were found to be positive for *bla_CTX-M-1_*/*bla_SHV_* gene. A total of 4 (10.14%) and 9 (2.17%) isolates were positive for *bla_CTX-M-1_* and *bla_SHV_* genes, respectively, whereas, 3 (0.72%) *K. pneumoniae* isolates were positive for both the genes. On the other hand, only 2 (0.48%) *Salmonella* isolates for *bla_CTX-M-1_* gene. Detail of the result is depicted in Table-3 and Figure 1.

**Table-3:** PCR-based detection of *bla_CTX-M-1_* and/or *bla_SHV_* genes in the bacterial isolates obtained from human beings in Mizoram.

Organisms	*bla_CTX-M-1_* (%)	*bla_SHV_* (%)	*bla_CTX-M-1_* and *bla_SHV_*
*E. coli* (n=333)	36 (8.70)	5 (1.21)	-
*K. pneumoniae* (n=52)	4 (0.97)	4 (0.97)	3 (0.72)
*Salmonella* spp. (n=29)	2 (0.48)	-	-
Total (n=414)	42 (10.14)	9 (2.17)	3 (0.72)

PCR=Polymerase chain reaction, *E. coli=Escherichia coli, K. pneumonia=Klebsiella pneumonia*, Figures in parenthesis are percentile values

**Figure-2:**
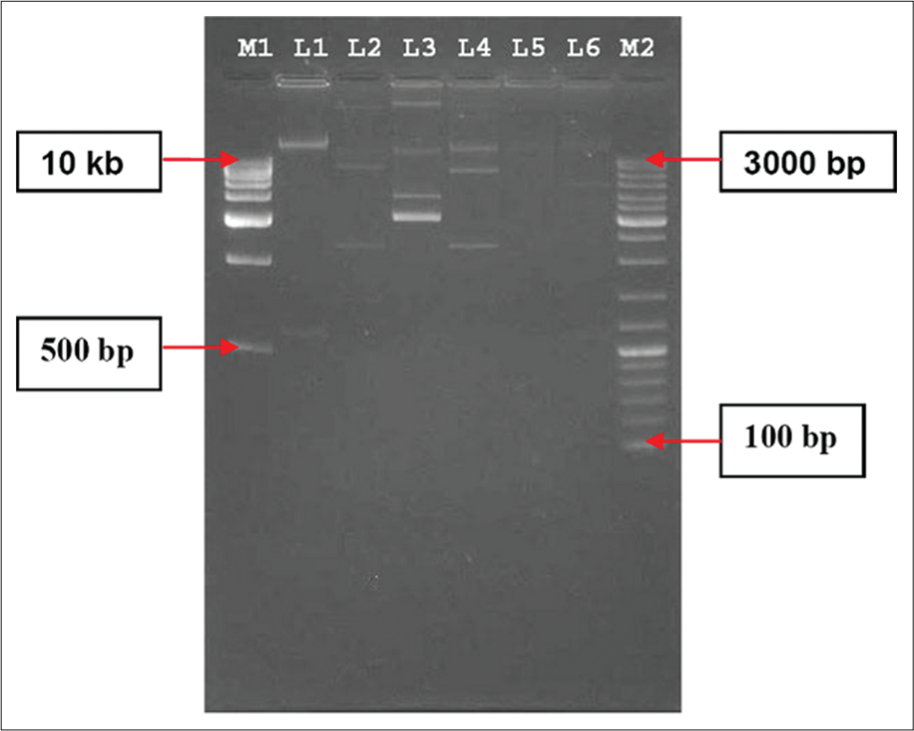
Agarose gel electrophoresis of plasmids extracted from the ESBLs positive isolates obtained from human beings in Mizoram. Lane M1: 1kb DNA ladder, Lane 1: Sample no. IKP-94, Lane 2: IEC-24, Lane 3: Sample no. IEC-46, Lane 4: Sample no. IEC-48, Lane 5: Sample no. IEC-49, Lane 6: Sample no. IKP-114, Lane M2: 100 bp DNA ladder. IEC: *Escherichia coli* isolates, IKP: *Klebsiella pneumoniae* isolates.

As mentioned in Figure 2, all the ESBLs producing isolates showed the presence of plasmids ranging from 0.5 kb to 30 kb. Using acridine orange (2-2.5 mg/ml), all the isolates were successfully cured (Table-4). Confirmation of curing was done by disc diffusion assay, where the organism showed 100% sensitivity against all the antibiotics; plasmid extraction could not trace any plasmids, and no ESBLs genes could be detected by PCR assay.

**Table-4:** Effect of acridine orange (2.0-2.5 mg/ml) mediated plasmid curing on drug resistance pattern of ESBLs positive isolates.

Antibiotics used	Diameter of zone of inhibition before curing (mm)				Diameter of zone of inhibition after curing (mm)			
	
	Isolates no				Isolates no			
	
	IEC-49 (1)	IEC-49 (2)	IEC-24	IKP-114	IEC-49 (1)	IEC-49 (2)	IEC-24	IKP-114
Cefixime	2	3	3	3	19	22	22	20
Cefotaxime	4	4	3	3	23	22	22	22
Ceftazidime	2	2	2	3	15	14	15	15
Ceftriaxone	4	4	4	3	23	25	22	25
Streptomycin	2	2	2	3	15	15	15	15

ESBLs=Extended spectrum β-lactamases, IEC=*Escherichia coli* isolates, IKP=*Klebsiella pneumoniae* isolates

By *in vitro* horizontal gene transfer assay, the resistance traits from any of the isolates could not be transferred to the recipient host.

## Discussion

In the present study, majority of the ESBLs producing isolates were *E. coli* (80.44%), followed by *K. pneumoniae* (12.56%) and *Salmonella* spp. (7.00%). Similar kind of results was also reported by other workers in India [8] and abroad [9]. Babypadmini and Appalaraju [10] also reported *E. coli* (57.8%) and *K. pneumoniae* (25.6%) as the major enteric bacterial population prevalent in the clinical samples of human beings in Chennai.

In antimicrobial sensitivity assay, the level of resistance to ceftriaxone, ceftazidime, and cefotaxime was found to be 94.89%, 100%, and 97.16%, respectively (data not shown). Our result is also in corroboration with the study conducted by Sasirekha *et al*. [11] and Singh and Goyal [12], where 84%, 75%, and 85% *E. coli* were reported to be resistant to cefotaxime, ceftriaxone, and ceftazidime, respectively. Similarly, in the present study, *Klebsiella kneumoniae* isolates were also found to be resistant to ceftriaxone, ceftazidime, and cefotaxime at the level of 100%, 100%, and 97.06%, respectively. Similarly, the *Salmonella* isolates were found to be resistant to ceftriaxone, ceftazidime, and cefotaxime at the level of 83.33%, 100%, and 83.33%, respectively. Similar kinds of observations were also reported by other workers [13]. This kind of high level of resistance to beta-lactam antibiotics by the *K. pneumoniae* isolates may be due to their virulence factor like hyperviscosity, polysaccharide capsule and production of endotoxin, and carbapenemase [12].

As mentioned in the present study, recovery of a higher (21.26%) level of ESBLs producing enteric bacteria are also reported by several other works in India, which varied from 6.6% to 91% [7,14]. The major reasons for the high prevalence of ESBLs producing organisms in human populations are may be because of indwelling catheters, endotracheal or nasogastric tube, gastrostomy or tracheostomy, severity of illness, and excessive use of third generation cephalosporins [7].

Approximately, 13.00% of the isolates were positive for the *bla_CTX-M-1_* and/or *bla_SHV_* genes, where *bla_CTX-M-1_* showed higher prevalence than its counterpart. Similar kind of trend has also been reported by Ensor *et al*. [15] and Jones *et al*. [16]. *CTX-M* may be increased due to the wide use of third generation cephalosporins, especially ceftriaxone and cefotaxime or may be associated with high mobilization of the encoding genes [17]. Barlow *et al*. [18] reported that the *bla_CTX-M_* genes have been mobilized to plasmids almost 10 times more frequently than other class A β-lactamases. A total of 8.70% *E. coli* isolates were found to be positive for *bla_CTX-M-1_* gene by specific PCR assay (Table-3 and Figure-1). Muzaheed *et al*. [19] also reported a high prevalence of CTX-M genes in *E. coli* from Southern India. The predominance of CTX-M type of ESBLs gene may be an indication that this allele would now be common in North-Eastern Region of India. On the other hand, only 1.21% *E. coli* and 0.97% *K. pneumoniae* were found to be positive for *bla_SHV_* gene. No *Salmonella* isolate was found to be positive for *bla_SHV_* gene. Similar type of finding was reported by Lin *et al*. [13] and Barguigua *et al*. [17] from a regional hospital in Central Taiwan and from community in Morocco. Rotimi *et al*. [20] reported that SHV gene was not detected in any of the *Salmonella* spp. isolated from fecal samples from Kuwait and UAE. As the members of *bla_SHV_* group are extremely diverse, and only one primer is included in the present study, some of our isolates may contain *bla_SHV_* genes not previously described.

A total of 3 (0.72%) *K. pneumoniae* isolates were recorded as positive for both *bla_CTX-M-1_* and *bla_SHV_* genes, but no *E. coli* or *Salmonella* spp. isolates were carrying both the genes. A similar kind of finding is also reported by Al-Agamy *et al*. [21], where they observed the presence of both *bla_CTX-M-1_* and *bla_SHV_* genes in 9.1% of ESBL producing *K. pneumoniae* from hospitalized patients in Saudi Arabia.

The prevalence of *bla_CTX-M_* producing *Salmonella* spp. was about 0.48%, which is also in corroboration with the reports of other workers. Li *et al*. [22] reported 1.5% CTX-M producing *Salmonella* spp. in Taiwan. The little variation of the results in this study in comparison to the other reports may be because of limited number of isolates used or lower expression of CTX-M genes due to overuse of 3GCs.

All the 4 selected isolates were cured using acridine orange and the subsequent plasmid extraction and PCR detection of target genes confirmed the curing effect (Table-4). Although, curing provides only the preliminary evidence that genetic traits are of extrachromosomal nature, the loss of growth on antibiotic containing plates shows that the multidrug resistance genes may be plasmid-borne. The resistance determining traits are often transposable, which exist in both plasmid and chromosomal locations (flip-flop) mechanism. It is, however, important to note that all antibiotic resistance genes are plasmid-mediated. Sometimes copies of the plasmids lying closer to the membrane are completely eliminated by curing agents, while those lying closer to the nucleus may escape the curing effect.

Plasmids extracted from the ESBL positive isolates were found to be within the range 0.5-30 kb. As depicted in Figure 2, the size of plasmids of different isolates under the study were variable, which is also in agreement with the observations by Akortha and Filgona [9], where they reported that plasmid of *K. pneumoniae* isolated from human patients are distributed widely with great diversity.

In the present study, 7 *E. coli* isolates, carrying *bla_CTX-M-1_* or *bla_SHV_* genes and resistant to multiple antibacterial drugs, were used as donor for *in vitro* trans-conjugation by horizontal gene transfer. Similarly, 1 *K. pneumoniae*, carrying both the genes and resistant to multiple antibacterial agents was used as donor for the same purpose. On the other hand, 2 *E. coli*, 3 *K. pneumoniae*, and 3 *Salmonella* spp. including ATCC 13076 were used as recipient. All the recipient isolates were sensitive to antibiotics and negative for both *bla_CTX-M-1_* and *bla_SHV_* genes. In case of all the donor isolates, the resistance determining genes were located in their plasmids. In conjugation study by broth mating, filter paper mating as well as plate mating method, neither of the plasmids carrying any one of the target gene could be transferred horizontally to the recipient isolates. A similarly low trans-conjugation success was also reported by other workers in Switzerland [23] and Germany [24].

Large plasmids can often mediate their own transfer from one cell to another by a process called conjugation. The occurrence of conjugative large plasmids in *K. pneumoniae* was reported by several investigators, who isolated *K. pneumoniae* strains harboring large plasmids encoding β- lactamases enzyme. In most cases, these plasmids were the conjugative type that encoded resistance to multiple antibiotics and metal ions. *In vitro* experiment showed transfer of the plasmids ranging from 108 kb to 157 kb, while *in vivo* conjugation experiment showed a transfer of smaller sized plasmids. Failure of conjugation in the present study may be because of the small sized plasmids present in the donors. In addition, conjugative isolates harbor the plasmids encoded *tra* genes encoding the transferase proteins TraM responsible for mating aggregation, Tra Y directing the nicking enzyme Tra T to Ori T, Tra I including nicking and unwinding and Tra D involved in pumping the DNA into the recipient cells [25].

## Conclusion

ESBLs producing *E. coli, Salmonella* spp. and *K. pneumoniae* were recorded from human beings in Mizoram. PCR analysis confirmed that 13.04% of the enteric bacteria of a human are harboring *bla_CTX-M-1_* and/or *bla_SHV_* gene, which were located in the plasmids. Although, the genes were not transferred horizontally by *in vitro* techniques, the possibility of transfer under selective pressure *in vivo* condition cannot be ruled out. More strict antibiotic policies are needed in order to prevent emergence and dissemination of these strains within the environment, animals, and humans.

## Authors’ Contributions

IW collected the samples, extracted nucleic acids, detected virulence genes, and ESBLs genes. TKD designed the program, analyzed the data, and prepared the manuscript. HL isolated the organisms, conducted antibiotic assays. RC monitored the program, prepared, and edited the manuscript.
